# Fine-tuning digital FIR filters with gray wolf optimization for peak performance

**DOI:** 10.1038/s41598-024-62403-6

**Published:** 2024-06-03

**Authors:** Anand R, Sathishkumar Samiappan, M. Prabukumar

**Affiliations:** 1 Department of Electrical and Electronics Engineering, Amrita School of Engineering, Coimbatore, Amrita Vishwa Vidyapeetham, India 641112; 2 Geosystems Research Institute, Mississippi State University, Starkville, MS USA; 3 School of Computer Science Engineering and Information Systems (SCORE), Vellore Institute of Technology, Vellore, 632014 India

**Keywords:** Evolution, Engineering, Mathematics and computing

## Abstract

The design of optimum filters constitutes a fundamental aspect within the realm of signal processing applications. The process entails the calculation of ideal coefficients for a filter in order to get a passband with a flat response and an unlimited level of attenuation in the stopband. The objective of this work is to solve the FIR filter design problem and to compare the optimal solutions obtained from evolutionary algorithms. The design of optimal FIR low pass (LP), high pass (HP), and band stop (BS) filters is achieved by the utilization of nature-inspired optimization approaches, namely gray wolf optimization ,cuckoo search, particle swarm optimization, and genetic algorithm. The filters are evaluated in terms of their stop band attenuation, pass band ripples, and departure from the anticipated response. In addition, this study compares the optimization strategies applied in the context of algorithm execution time which is achievement of global optimal outcomes for the design of digital finite impulse response (FIR) filters. The results indicate that when the Gray wolf algorithm is applied to the development of a finite impulse response (FIR) filter, it produces a higher level of performance than other approaches, as supported by enhanced design precision, decreased execution time, and achievement of an optimal solution.

## Introduction

Filtering is typically the most intricate procedure employed in signal processing. In digital signal processing, filters change the spectrum of the input signal so that the output signal has the right spectrum properties. Digital filters are widely used because they have linear phase characteristics, work accurately, are stable at high temperatures, can be changed with a programmable processor, can multiplex, log data, and can be used over and over again. They can work with both real-time and stored data and can be built in both hardware and software^1^. There are two types of digital filters: finite impulse response (FIR) filters and infinte impulse response (IIR) filters. If there is no need for phase shift, FIR filters are the best choice to choose. FIR filters are also naturally stable systems that aren’t as affected by the limited word length effect. On the other hand, IIR filters have fewer factors, use less memory, and are used when there needs to be a sharp cut-off^2^. Unlike the design of IIR filters, the design of FIR filters is not related to the design of analogue filters. The design of FIR filters is primarily focused on directly approximating the given magnitude response, while also typically requiring the phase response to be linear. The transfer function *H*(*z*) of length $N+1$ is a causal Finite Impulse Response (FIR) polynomial of degree N, where $z^{-1}$ represents the backward shift operator is shown in Eq. (1).

A sequence *x*(*n*) with a finite duration and length N+1 can be fully described by N+1 samples of its discrete Fourier transform $X(e^{-jw})$. Therefore, it is feasible to create a FIR filter by determining N+1 samples of its frequency response or impulse response h(n), resulting in a filter length of N+1. The conditions for the linear phase design are as follows:

1 $$\begin{aligned} h(n)=\pm h(N-n) \end{aligned}$$

Researchers have devised and implemented heuristic evolutionary optimization algorithms^3^ in accordance with the principles of natural selection and evolution. The genetic algorithm optimization (GAO), which was introduced in 1975, is a class of probabilistic search algorithms designed for general purposes. It draws inspiration from natural genetic populations and aims to evolve solutions to optimization and search problems with a high degree of success. Substantial effort has been dedicated to the construction of optimum filters using GAO and its variations^4^. PSO has the ability to handle functions that are not differentiable and have many objectives^5,6^ and guarantees global solutions. Developed in 1996, differential evolution (DE) is an additional stochastic, population-based optimization technique^7,8^. Methods for designing FIR filters utilizing the DE algorithm are detailed in reference^9^. An additional bio-inspired algorithm is cuckoo search optimization (CSO), which was introduced in 2007 and is more versatile by incorporating the intelligent foraging behavior and intriguing characteristics of a honey bee swarm in its quest for sustenance^10,11^. Cuckoo-search optimization (CSO) is one of the most recent meta heuristic optimization techniques inspired by nature; it was created in 2009^12,13^. Recent research suggests that among several nature-inspired algorithms, CSO is the most efficient optimization tool for solving structural engineering challenges^14,15^.

Mirjalili et al.^16^ introduced the GWO, a novel meta heuristic algorithm that emulates the natural hunting mechanism and social hierarchy of grey wolves. The algorithm consists of three primary stages: encircling prey, pursuing prey, and attacking prey. The mathematical representation of the leadership hierarchy of wolves designates the ideal option as alpha, with the second and third optimal alternatives being represented as delta and beta, respectively. The remaining possible solutions are assumed to be omega^17^.

An exhaustive and integrated examination of GWO is utilized to design a FIR LP, HP, and BS filter in this article. By utilizing a comparable design methodology, it is possible to modify these filters to create a additional FIR filters like band pass filter. The process of developing an ideal filter entails determining the filter coefficients that generate a passband ripple response of minimal magnitude and a stopband attenuation that is of significant magnitude. This is determined by the coefficients that are computed using GWO^17^. A comparative analysis is conducted between the designs and alternative bio-inspired optimization techniques, namely PSO, CSO, and GAO.

The structure of this research work as follows. Section refs2 delineates the mathematical formulation of the problem pertaining to the design of the FIR filter with cost function estimation. The algorithms utilized in the design of the FIR filter are the subject of Section refs3, which also describes the implementation of these algorithms that are specific to the problem at hand. Section refs4 describes the results and analysis of the simulations that were conducted. Section refs4 provides a detailed analysis of the effectiveness of FIR filters and an update on the progress of GWO in compared to other algorithms. Section refs5 provides the concluding remarks for the entire endeavor.

## Classification of finite discrete length sequences

### Derived from the principles of geometric symmetry

Geometric symmetry is a significant factor in digital signal processing applications, as evidenced by the utilization of finite discrete samples. There are two types of geometric symmetric are used: (i) N-point symmetric and (ii) N-point anti-symmetric^18,19^. The following condition should be satisfies for a length N-point symmetric response:

2 $$\begin{aligned} x[n]=x[N-1-n] \end{aligned}$$

**Figure 1:**
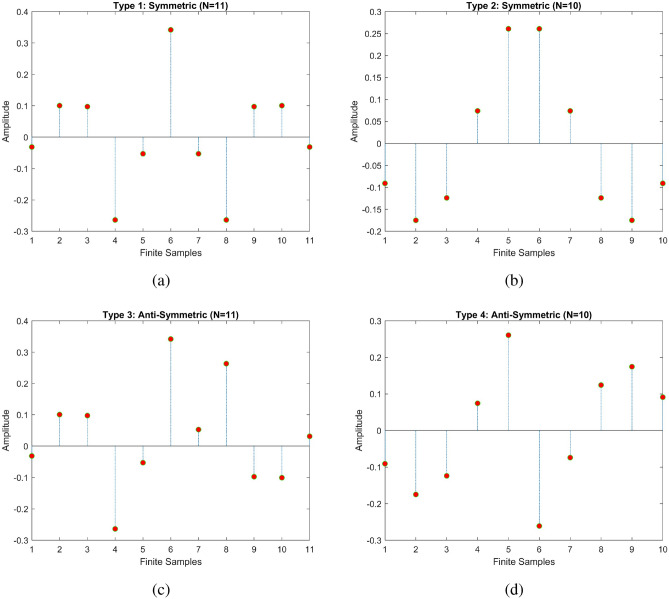
(**a**) Type 1: Order =11 (**b**) Type 2: Order=10 (**c**) Type 3: Order=11 (**d**) Type 4: Order=10.

The following condition should be satisfies for a length N-point anti-symmetric response:

3 $$\begin{aligned} x[n]=-x[N-1-n] \end{aligned}$$

where N can be either even or odd samples, based on N values the 4 different types of geometric symmetric condition are formed. Figure 1 shows the four types of geometric symmetry with center for symmetry.

From the Fig. 1, In the cases of type 1 and type3 sequences, the point of symmetry lines up with one of the samples in those sequences. This is why it is called “whole-sample symmetry.” For type 2 and type 4 sequences, on the other hand, the point of symmetry is in the middle of two center samples, which is why this type of symmetry is called “half-sample symmetry.” Thus, sequences of types 1, 2, 3, and 4 are called whole sample symmetric, half sample symmetric, whole sample anti-symmetric, and half sample anti-symmetric, respectively^20^.

#### Type 1: symmetric sequence with N=odd

Type 1 linear phase odd length sequence, the corresponding Fourier transform of the sequence is shown in Eq. (4),

4 $$\begin{aligned} X[e^{jw}]=e^{-{j(N-1)\frac{\omega }{2} }}\begin{Bmatrix} X[\frac{N-1}{2}] +2 \times \sum _{n=1}^{\frac{N-1}{2}} x(\frac{N-1}{2}-n)cos(\omega n) \end{Bmatrix} \end{aligned}$$

#### Type 1: symmetric sequence with N=even

Type 2 linear phase even length sequence, the corresponding Fourier transform of the sequence is shown in Eq. (5),

5 $$\begin{aligned} X[e^{jw}]=e^{-{j(N-1)\frac{\omega }{2} }}\begin{Bmatrix} 2 \times \sum _{n=1}^{\frac{N}{2}} x(\frac{N}{2}-n)cos(\omega (n-\frac{1}{2})) \end{Bmatrix} \end{aligned}$$

#### Type 3: anti-symmetric sequence with N=odd

Type 3 linear phase even length sequence, the corresponding Fourier transform of the sequence is shown in Eq. (6),

6 $$\begin{aligned} X[e^{jw}]=j \times e^{-{j(N-1)\frac{\omega }{2} }}\begin{Bmatrix} 2 \times \sum _{n=1}^{\frac{N-1}{2}} x(\frac{N-1}{2}-n)siin(\omega (n)) \end{Bmatrix} \end{aligned}$$

#### Type 4: anti-symmetric sequence with N=even

Type 4 linear phase even length sequence, the corresponding Fourier transform of the sequence is shown in Eq. (7),

7 $$\begin{aligned} X[e^{jw}]=j \times e^{-{j(N-1)\frac{\omega }{2} }}\begin{Bmatrix} 2 \times \sum _{n=1}^{\frac{N}{2}} x(\frac{N}{2}-n)siin(\omega (n-\frac{1}{2})) \end{Bmatrix} \end{aligned}$$

Consequently, the FIR filter design error function possesses L+2 or L+3 extrema, where L denotes the greatest limit of symmetry. Usually, extra ripple (side lobe) filters are those that have more than L+2 changes or ripples in their form. We can raise the number of the filter to get rid of the ripples. But as the order goes up, it may take longer and be more difficult to build the structures. So, we came up with a new way to cut down on the ripples and improve the performance of the FIT filter design using meta-heuristic optimizations^21,22^.

### Cost function estimation for high pass and band pass FIR filter

In this paper, we used^23^ cost function for high pass filter is modified as follows^24^:

8 $$\begin{aligned} \phi =\alpha \times E_p+(1-\alpha ) \times E_s \end{aligned}$$

where, $\alpha =\frac{N-1}{2}$ , $E_p$ and $E_s$ is the pass and stop band error for low pass FIR filter which can be formulated by [];

9 $$\begin{aligned} E_s=b^TCb \end{aligned}$$

10 $$\begin{aligned} E_p=\frac{\omega _p}{\pi }-2 \times b^TP+b^TQb \end{aligned}$$

where, b is the symmetric sequences, stop band error has two coefficients P and Q are to be calculated by:

11 $$\begin{aligned} P=\frac{1}{\pi }\int _{0}^{\omega _p} cos(\alpha \omega ) d\omega \end{aligned}$$

12 $$\begin{aligned} Q=\frac{1}{\pi }\int _{0}^{\omega _p} cos(\alpha \omega -n) sin(\alpha \omega -n)d\omega \end{aligned}$$

In case of pass band error C are calculated by:

13 $$\begin{aligned} C(m,n)=\frac{1}{\pi }\int _{w_s}^{\pi } cos(\alpha \omega -n) sin(\alpha \omega -n)d\omega \end{aligned}$$

In case of band stop filter, the FIR filter cost function can be defined as:

14 $$\begin{aligned} \phi =\alpha \times E_p+(1-\alpha ) \times (E_{s1}+E_{s2}) \end{aligned}$$

where, $E_{s1}$ and $E_{s2}$ as same as discussed previous, only limit has to consider for the same. Here, the goal of this work is to reduce the side lobe and reduce the transition band^25^.

### Grey wolf optimization

Grey wolf optimization (GWO) is a meta-heuristic algorithm proposed initially in 2014 by Mirjaliali^16^. Grey wolves employ this method of locating the optimal solution through the use of social hierarchy and foraging strategies^17^. This grey wolf has the advantages of being loyal to pack members, working together in a pack, and being a dominant pack leader. The wolf helps to find the decision-making of the optimal bands. GWO algorithm mimics the leadership and hunting mechanism of grey wolves which is shown in algorithm 1.

The Mathematical model of GWO algorithm is discussed in following steps: (i) Convergence of social hierarchy and hunting strategy. (ii) Find fitness solution of Alpha $(\alpha )$ wolf. (iii) Find the second and third best fit solution, Beta $(\beta )$ and Delta $(\delta )$ wolves respectively. (iv) Finally, Omega $(\omega )$ of these three wolves^16^. Grey Wolf encircle the prey during hunting and so, encircling model behavior is shown in Eqs. (15) and (16),

15 $$\begin{aligned} D=|\ C \times X_p\ -X(t)| \end{aligned}$$

16 $$\begin{aligned} X(t+1)=X_p\ (t)-A \times D \end{aligned}$$

where, t is the iteration, $\left\{ X_p\right\}$ is location of the pray, $\left\{ X\right\}$ is the wolf position location and $\left\{ A\right\} \,\ \left\{ C\right\}$ are coefficient vectors. It has the value of $A\ =2 \times a \times \left\{ r_1\right\} -a$ and $C\ =2 \times r_2$ respectively. Here $\left\{ r_1\right\} , \left\{ r_2\right\}$, are random vectors that range from 0 to 1 and The component exhibits a linear drop from 2 to 0 during the iterations, enabling the wolf to attain any location within the range of the two spots^14^.

**Algorithm 1:**
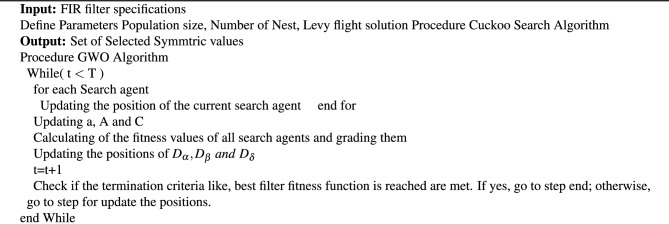
FIR filter design using GWO.

**Figure 2:**
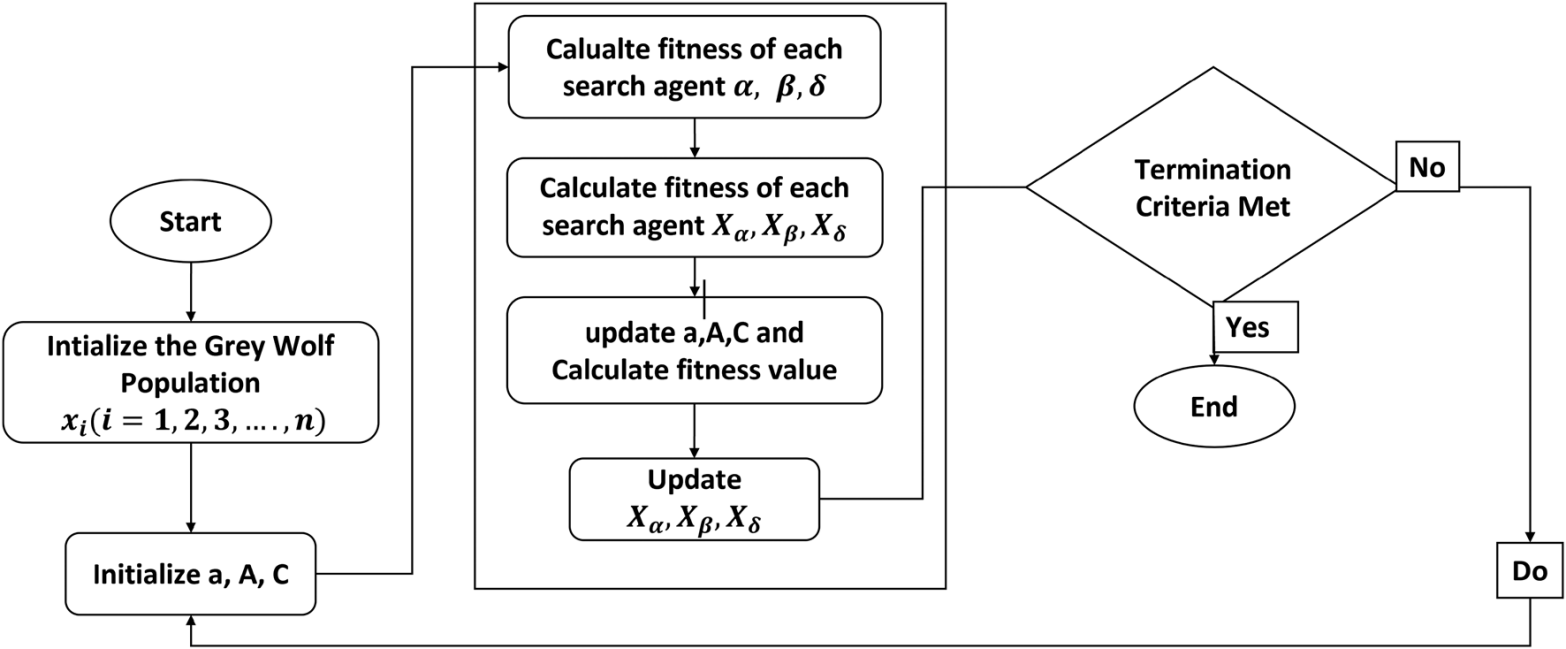
Flow chart of GWO.

Finally, the position of the grey wolves is shown in Eq. (17), and the flow chart of GWO optimization is shown in Fig. 2.

17 $$\begin{aligned} X(t+1)=\frac{(X_1+X_2+X_3)\ }{3} \end{aligned}$$

## Proposed methodology

A summary is provided of the procedures followed in order to address the filter design problem using gray wolf optimization (GWO). In the process of designing FIR LP, HP, and BS filters, respectively, the error target functions (fitness functions) specified in Eqs. (6) and (7) are utilized to evaluate each potential solution. To make a Nth order FIR filter, the best way to solve this problem is to get N+1 symmetric filter coefficients. This is made sure of by setting the symmetry condition on the coefficients that need to be improved. For symmetric coefficients, $h(n)=h(N-1-n)$, and $h(n)=-h(N-1-n)$ for anti-symmetric coefficients. The range of values for n is 0 to N. This means that the filter that was built has a linear phase response^2^.

### Filter design using GWO

**Figure 3:**
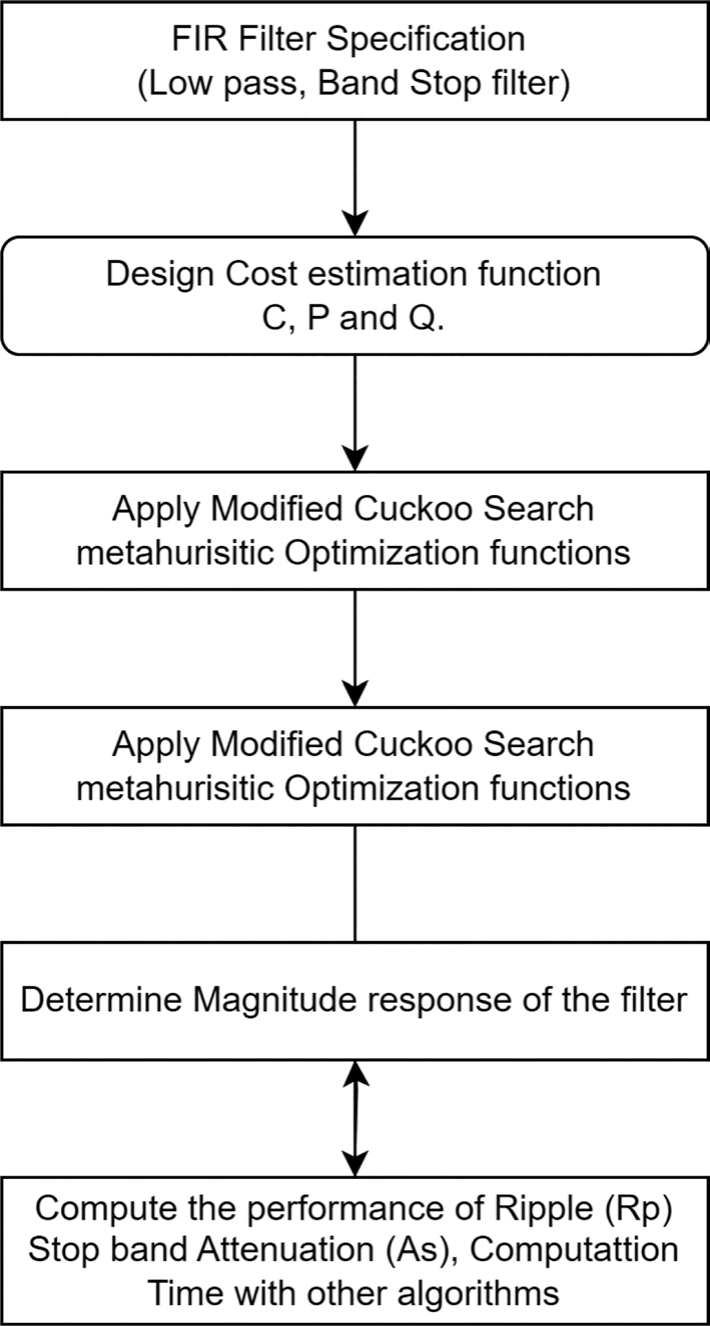
Flow chart of proposed methodology.

The subsequent procedures for implementing the Grey Wolf Optimization (GWO) algorithm for the design challenges of Finite Impulse Response (FIR) filters are outlined. The equations represent the objective functions of the cost estimation, as indicated by Eqs. (8) and (14), which are assessed at each iteration for the potential solutions in the construction of FIR filters. The adaptive values of a and A determine the parameter values of gray wolf population and exploration and exploitation. These two factors are dynamically adjusted and facilitate a seamless transition between exploration and exploitation. When decreasing the value of A, the first half of the values will be dedicated to exploration, while the remaining values will be focused on exploitation. The proposed flow are described in the Fig. 3. There will be only two ways to improve the GWO performance by adjusting a and C. The following steps are to followed to find the optimal FIR filter coefficients.

Step 1: Augment the gray wolf population by 100, a= [0, 1], c= [0, 2] and A= [0, 1] and maximum objective function is fixed as − 1. +1 for LPF and BSF. We assume that number of iterations as N=4000.
Step 2: Assume an initial population randomly, where each individual represents a set of variable filter coefficients x=[x0, x1, x2…xN]. The variable x denotes the collection of N+1 filter coefficients that are to be improved.
Step 3: Calculate the gray wolf fitness functions of the wolves that were randomly created initially, as well as the objective function in Eqs. (8) and (14) at iteration i.
Step 4: Produce a novel solution utilizing the hunting position model as depicted in the Eqs. (9) and (10).
Step 5: Remove the wolves with the lowest fitness values based on the probability and create a new wolf.
Step 6: Calculate gray wolf the fitness functions of all the new wolves and update the best wolves (best filter coefficient).
Step 7: Continue executing steps 2 to 6 iterative until the last iterations of the cost function, resulting in the acquisition of the best wolves and their corresponding optimal filter coefficients, denoted as x.

The process of configuring algorithm control parameters is a formidable challenge. The nature of the problem at hand renders it an optimization problem, and the algorithm’s efficacy might be significantly impacted in relation to the specific problem being addressed. In the existing body of literature, there is a lack of a specific and clearly defined approach for the task at hand. The process of optimizing parameters. Researchers conducted comprehensive simulations using several sets of parameter values as indicated in their study. The concept of “range” refers to the extent or scope of something. It is often This paper use a similar methodology^13^. Conducting numerous simulations with minor adjustments to the values of the control parameters, while adhering to the specified range indicated in the study. According to the literature^18^, in this study, the selection of parameters for each algorithm is conducted subsequent to thorough analysis and evaluation. Several simulations were conducted using a range of values as described in the previous research. The filter design method is outlined in Table 1.

**Table 1:** Parameters for controlling the design of a finite impulse response (FIR) filter.

Parameters	Symbol	PSO	CSO	GA	MFO	WOA	BBA	GWO
Population size	dim	100	100	100	100	100	100	100
Iteration	N	4000	4000	4000	4000	4000	4000	4000
Filter coefficients		− 1, +1	− 1, +1	− 1, +1	− 1, +1	− 1, +1	− 1, +1	− 1, +1
Parameters	C1, C2 for PSO A, C for CSO $\alpha , \beta , \delta$ for GWO	2, 2	2, 2		2, 2	2, 2	2, 2	2, 2
Particle velocity	$v_{min}$, $v_{max}$	0.01, 0.2	–	–				–
Inertia weight	W	0.2	–	–		0.2		–
Discovering rate	$P_a$	–	0.25	–	0.25		0.25	0.5
Mutation rate	Adaptive value			0.01				2

In the context of genetic algorithms (GAO), the selection process is carried out by employing a tournament operator with a size of 6. This approach enhances the effectiveness of the selection process by providing a selection pressure, so enabling the identification of the most optimal individual. The recommended crossover probability falls within the range of 0.6 to 0.95. In this particular case, it has been set to 0.8. This choice is based on the observation that the algorithm consistently converged within a significant number of iterations when the crossover probability was below this value. Conversely, increasing the crossover rate resulted in premature convergence. The mutation probability varies between 0.001 and 0.05. The solution exhibits reduced disturbance when low values are utilized, whereas a high mutation rate provides increased diversity. In this study, a fixed mutation rate of 0.01 is employed to augment the capacity for exploration. In order to achieve efficient exploration performance, PSO algorithm parameter are typically adjusted to a value of 2. The observed range of particle velocity is determined to be within the interval [0, 1]. The higher success rate of CSO can be attributed to its reliance on a limited number of parameters. The study reveals that no fine-tuning is necessary, and the CSO method is not affected by any modifications in the parameters pa and dimensions^14^. When pa is equal to 0.25, a set portion of one-fourth of the time is allocated for the exploitation process, while the remaining three-fourths are dedicated to exploring the search space in pursuit of a global solution. This ensures the fulfillment of the global optimal criterion with a greater likelihood. For GWO, mutation rate is fixed on 2 based on literature^13^ with discovering rate of 0.5. In terms of learning parameters, A is between [0,2] and C value fixed by 1. For all the case, limits of filter coefficients are fixed, − 1, +1 for LPF and BSF respectively.

## Experimental Results

The evaluation of the performance of the FIR filters that have been built using various evolutionary algorithms (EA) is conducted through the analysis of the filter function, efficiency, and the execution time of the algorithms. The simulations were performed using MATLAB 2023A on a computer system featuring an Intel Core i3 CPU operating at a frequency of 2.3 GHz and 8 GB of RAM. The results presented in this study were derived from an extensive series of approximately 30 simulation trials, wherein the parameters were randomly altered. To ascertain the efficacy of the optimally constructed filter via Grey Wolf Optimization (GWO) and other meta heuristic optimization techniques, we also adopt the standard Parks McClellan’s (PM) technique, which is commonly utilized for designing equiripple filters, for the sake of comparison. The collection of solutions in which the lowest fitness value is attained is documented as the optimal solution. Table 1 presents the governing parameters for the four algorithms, namely CSO, PSO, GA, and GWO^13^.

### Low pass FIR filter

The following are the FIR design specifications: The filter order is represented by N = 18 and 19, respectively, and the cut-off frequency is indicated by $\omega _c$ $= 0.34$. The cost objective function for the algorithms employed in this work is denoted by Eqs. (1) to (4). The derived optimal filter coefficients for the 20th and 21st order FIR) Low pass filter utilizing Particle Swarm Optimization (PSO), Cuckoo Search Optimization (CSO), Genetic algorithm Optimization (GAO), and Grey Wolf Optimization (GWO) approaches are described from Tables 2, 3 respectively. Table 4 shows the qualitative analysis for 21st order low pass FIR filter and compared with 6 different metaheuristic optimization algorithms.

**Table 2:** For N=18, 19th order coefficients with symmetric values.

Optimization Coefficients	PSO	CSO	GAO	GWO
h(0)=h(19)	0.014325	− 0.00926	− 0.00257	0
h(1)=h(18)	− 0.02002	0.01698	0.01406	0.00165
h(2)=h(17)	− 0.02022	0.02894	0.03078	0.00736
h(3)=h(16)	0.01091	− 0.02545	− 0.02801	− 0.01010
h(4)=h(15)	0.00244	− 0.00845	− 0.00916	− 0.00449
h(5)=h(14)	− 0.08648	0.40421	0.42748	0.26741
h(6)=h(13)	− 0.17510	1.02628	1.06199	0.80239
h(7)=h(12)	− 0.12731	0.87674	0.89216	0.77428
h(8)=h(11)	0.07381	− 0.56386	− 0.56739	− 0.53934
h(9)=h(10)	0.26580	− 2.13600	− 2.13748	− 2.12551

**Table 3:** For N=19, 20th order coefficients with symmetric values.

Optimization Coefficients	PSO	CSO	GAO	GWO
h(0)=h(18)	0.10568	0.03232	0	1.32106
h(1)=h(17)	− 0.03585	− 0.03179	− 0.00380	− 0.33290
h(2)=h(16)	− 0.15768	− 0.17322	− 0.04275	− 0.84045
h(3)=h(15)	0.00486	0.00542	0.00204	0.01570
h(4)=h(14)	− 0.39200	− 0.42598	− 0.21980	− 0.85196
h(5)=h(13)	1.29051	1.36261	0.89864	2.08189
h(6)=h(12)	1.48673	1.53286	1.2164	1.93082
h(7)=h(11)	− 4.50701	− 4.56827	− 4.12587	− 5.05055
h(8)=h(10)	− 0.98409	− 0.98740	− 0.96270	− 1.01217
h(9)	6.47714	6.47715	6.47714	6.47714

**Table 4:** Qualitative analysis for 21st order low pass FIR filter.

Algorithm	Minimum stopband attenuation (db)	Minimum stopband attenuation	Execution time (sec)
GWO	− 33.47	0.03	0.374
PSO	− 20.37	0.57	3.1518
GAO	− 16.57	0.36	3.414
CSO	− 16.24	0.79	0.4236
MFO	− 22.41	0.45	0.524
WOA	− 19.47	0.09	0.914
BBA	− 24.79	0.37	1.754

**Figure 4:**
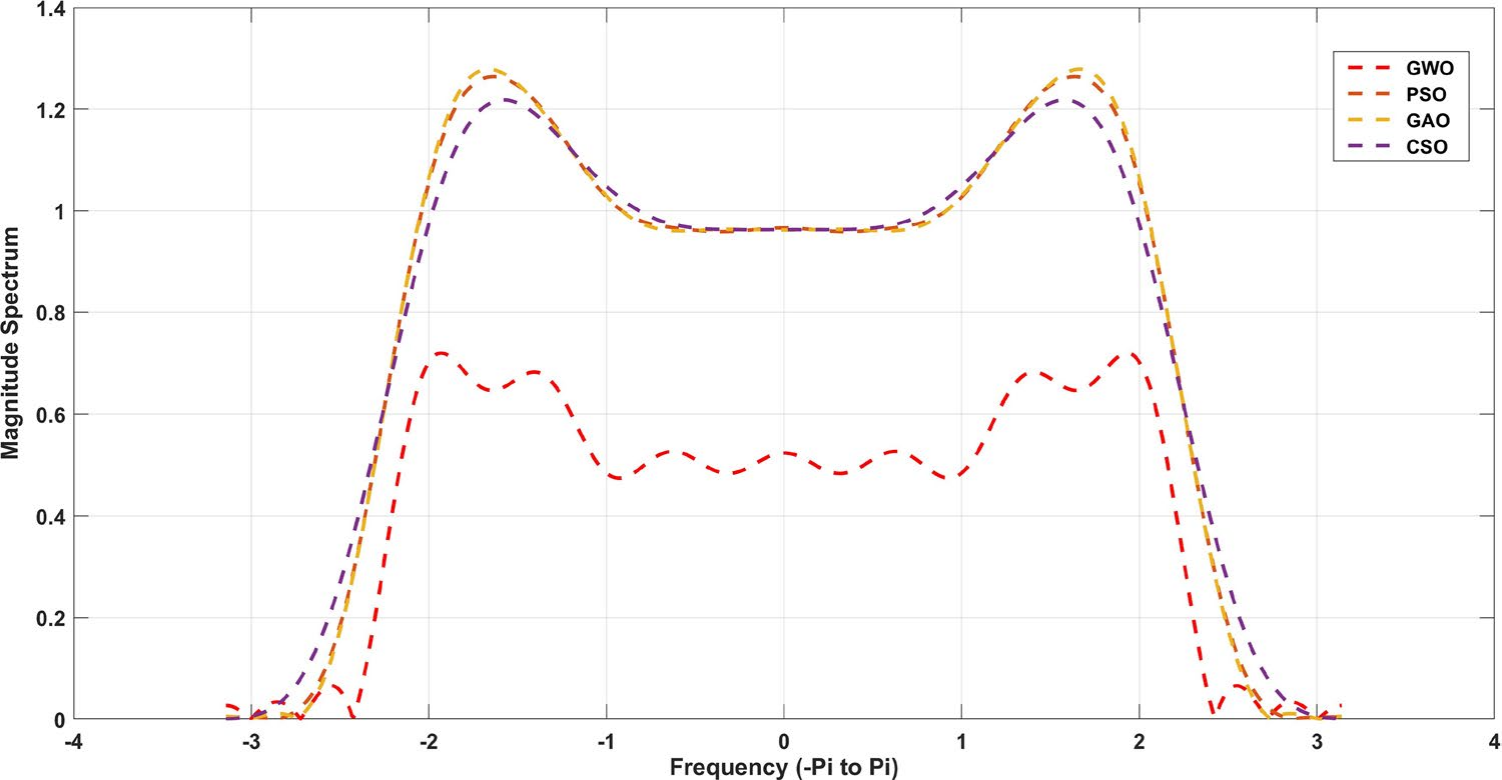
Magnitude response for the 21st order FIR Low pass filter.

**Table 5:** Qualitative analysis for 20th order low pass FIR filter.

Algorithm	Minimum stopband attenuation (db)	Minimum stopband attenuation	Execution time (sec)
GWO	− 36.59	0.03	0.371
PSO	− 16.06	0.46	3.471
GAO	− 14.40	0.30	3.14
CSO	− 12.20	0.94	0.4214
MFO	− 17.41	0.14	0.471
WOA	− 20.85	0.47	0.547
BBA	− 12.43	0.65	1.987

The Fig. 4 for 20th order and 5 for 21st order shows a graphical comparison of the magnitude response of the designed FIR low pass filter. The plot clearly demonstrates that GWO possesses the highest capacity to reduce signal strength in the stopband region. The filters are defined principally by their levels of lowest stop band attenuation (Astop) and maximum passband attenuation (Apass). These measures are given in Table 4 for 20th order and 5 for 21st order FIR low pass filters. From this Table 4,It has been discovered that a GWO produces the lowest stopband attenuation of − 36.59dB in comparison to PSO (− 16.06dB), GAO (− 14.40dB) and CSO (− 12.20dB). However, the maximum passband attenuation of GWO is 0.03dB, which is minimum from all the other method and same for Table 5 also. In terms of execution time, it is taking very less time to compute the filter coefficients compared to other methods. This is due to the GWO algorithm has better computing performance and it has capability to work together in a pack of symmetry coefficients with very less convergence rate.

**Figure 5:**
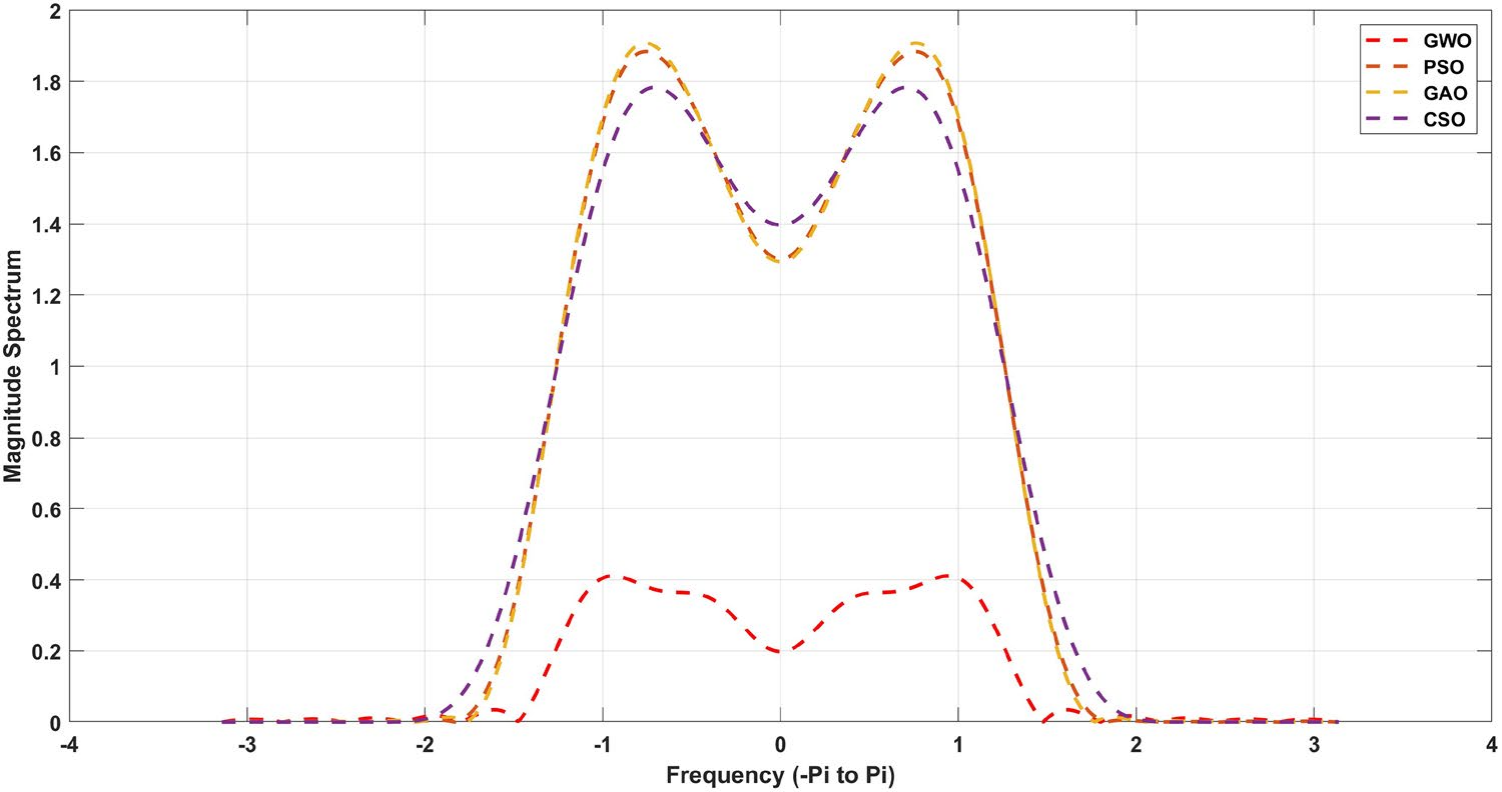
Magnitude response for the 20th order FIR Low pass filter.

The stopband attenuation coefficients of the developed FIR low pass filter are presented in Tables 6 and 7. As seen in Table 6, the average value of the GWO based FIR low pass filter is significantly low, which indicates the consistent performance of the stopband region. The same observation applies to Table 7. The magnitude response of the suggested filter is depicted in Figs. 4 and 5. From these figures, GWO shows better performance with very less discontinuity of the FIR filter whereas PSO, GAO and CSO has the moderate discontinuity and almost all the optimization has same magnitude of overshoot. The maximum normalized passband ripple of FIR low pass filter of GWO is 0.0129 for 21st order filter and 0.0769 for 20th order filter, which makes a overshoot of 0.0041% above the ideal response.

**Table 6:** Quantitative analysis for 21st order low pass FIR filter.

Algorithm	Maximum	Mean	Variance	Standard deviation
GWO	0.0128	0.0108	0.0001	0.0003
PSO	0.0891	0.0748	0.0005	0.0020
GAO	0.0931	0.0782	0.0005	0.0021
CSO	0.0783	0.0658	0.0004	0.0017
MFO	2.5484	2.1407	0.0145	0.0563
WOA	0.1405	0.1180	0.0008	0.0031
BBA	1.0931	0.9182	0.0062	0.0241

**Table 7:** Quantitative analysis for 20th order low pass FIR filter.

Algorithm	Maximum	Mean	Variance	Standard deviation
GWO	0.0796	0.0669	0.0005	0.0018
PSO	0.0513	0.0431	0.0003	0.0011
GAO	0.0546	0.0458	0.0003	0.0012
CSO	0.0429	0.0360	0.0002	0.0009
MFO	2.9401	2.4697	0.0167	0.0649
WOA	0.0748	0.0628	0.0004	0.0017
BBA	1.0546	0.8858	0.0060	0.0233

### High pass FIR filter

The design specifications are outlined as follows: The filter order is denoted as N = 18 and 19, while the cut-off frequency is represented as $\omega _c$ $= 0.34$. The cost objective function for the FIR high filter design algorithms utilized in this study is represented by Eqs. (1) to (4). The function is assessed at each iteration to find an optimal solution 8.

**Figure 6:**
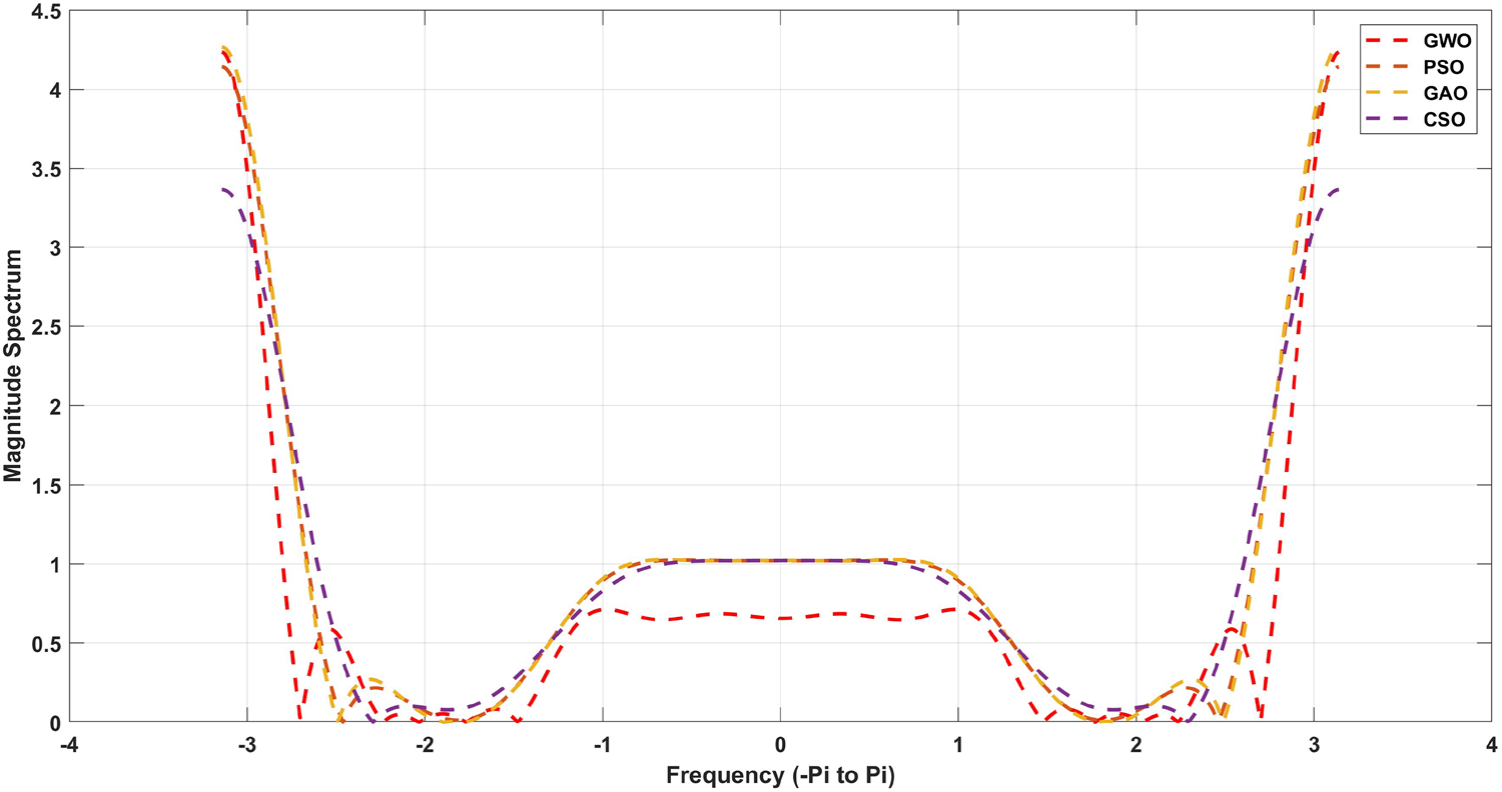
Magnitude response for the 21st order FIR High pass filter.

**Figure 7:**
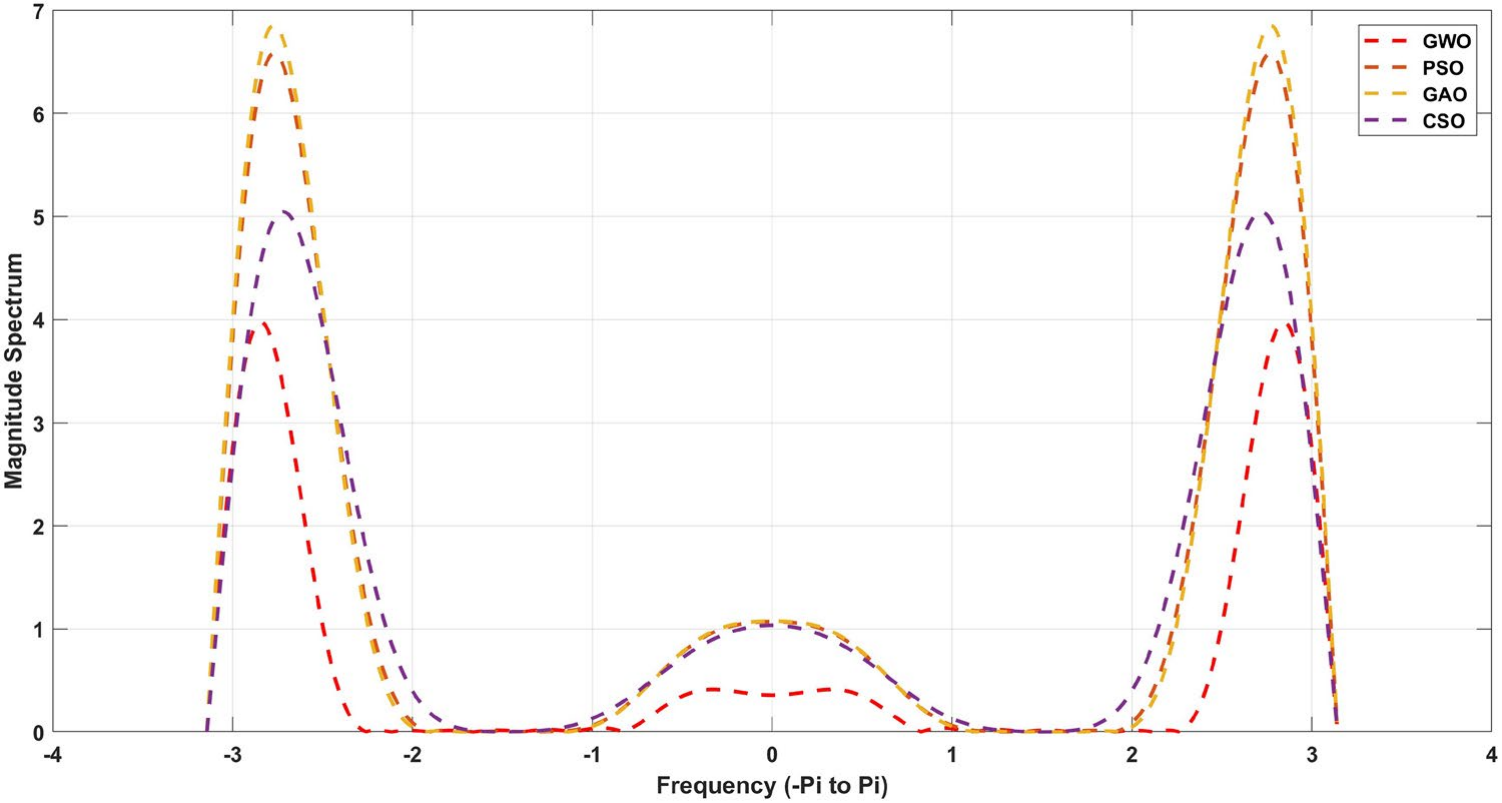
Magnitude response for the 20th order FIR High pass filter.

**Table 8:** Qualitative analysis for 21st order high pass FIR filter.

Algorithm	Minimum stopband attenuation (db)	Minimum stopband attenuation	Execution time (sec)
GWO	− 39.10	0.04	0.4124
PSO	− 31.75	0.40	2.5518
GAO	− 30.80	0.26	3.169
CSO	− 30.02	0.57	0.4936
MFO	− 30.67	0.47	0.879
WOA	− 27.67	0.85	1.245
BBA	− 26.58	0.94	1.4587

**Table 9:** Qualitative analysis for 20th order high pass FIR filter.

Algorithm	Minimum stopband attenuation (db)	Minimum stopband attenuation	Execution time (sec)
GWO	− 31.80	0.07	0.3452
PSO	− 6.58	0.46	2.917
GAO	− 7.66	0.38	3.894
CSO	− 5.40	0.97	0.3961
MFO	− 18.41	0.54	1.414
WOA	− 21.2	0.29	1.57
BBA	− 8.54	0.65	1.987

The Figs. 6 and 7 clearly describes that GWO has the maximum ability of attenuating signal in stopband region. These measures are given in Table 8 for 20th order and 9 for 21st order FIR high pass filters. From this Table 9, its observed that a GWO yields the minimum stopband attenuation of − 31.80dB in comparison to PSO (− 6.58dB), GAO (− 7.66dB) and CSO (− 5.40dB). However, the maximum passband attenuation of GWO is 0.07dB, which is minimum from all the other method and same for Table 8 also. In Tables 10 and 11 shows a stopband attenuation coefficients of the designed FIR high pass filter. As indicated in Table 10, the mean value of the GWO based FIR high pass filter is significantly low, which indicates the consistency of the performance in the stopband region. The same observation applies to Tables 4, 7. The magnitude response of the developed filter is depicted in Figs. 6 and 7. Based on the data, it can be observed that GWO exhibits superior performance with little discontinuity in the FIR filter. On the other hand, PSO, GAO, and CSO have moderate levels of discontinuity. Additionally, all optimization methods exhibit similar levels of overshoot. The FIR high pass filter of GWO has a maximum normalized passband ripple of 1.8343 for a 21st order filter and 0.04378 for a 20th order filter. This results in an overshoot of 0.0037% above the optimal response.

**Table 10:** Quantitative analysis for 21st order high pass FIR filter.

Algorithm	Maximum	Mean	Variance	Standard deviation
GWO	1.8343	1.5408	0.0104	0.0405
PSO	2.0508	1.7227	0.0116	0.0453
GAO	2.1061	1.7691	0.0120	0.0465
CSO	1.3803	1.1594	0.0078	0.0305
MFO	2.9258	2.4577	0.0166	0.0646
WOA	2.5807	2.1678	0.0146	0.0570
BBA	3.1061	2.6091	0.0176	0.0686

**Table 11:** Quantitative analysis for 20th order high pass FIR filter.

Algorithm	Maximum	Mean	Variance	Standard deviation
GWO	0.0438	0.0368	0.0002	0.0010
PSO	0.0495	0.0416	0.0003	0.0011
GAO	0.0508	0.0427	0.0003	0.0011
CSO	0.0625	0.0525	0.0004	0.0014
MFO	1.8924	1.5896	0.0107	0.0418
WOA	0.1099	0.0923	0.0006	0.0024
BBA	1.0508	0.8827	0.0060	0.0232

### Band stop FIR filter

The Band stop FIR filter designs specification are as follows, filter order will be 20th and 21st, the cut-off frequency was fixed $\omega _{c1} =0.35\pi$ and $\omega _{c2} =0.73\pi$. The objective fitness function of GWO is defined Eqs. (13) and (14). The optima filter coefficients are limited to a maximum of +1 and minimum of − 1.

**Table 12:** Qualitative analysis for 21st order band pass FIR filter.

Algorithm	Minimum stopband attenuation (db)	Minimum stopband attenuation	Execution time (sec)
GWO	− 28.645	0.05	0.4714
PSO	− 20.372	0.43	2.524
GAO	− 15.045	0.28	3.424
CSO	− 17.521	0.91	0.4956
MFO	− 20.98	0.55	0.948
WOA	− 29.12	0.14	1.414
BBA	− 32.44	0.77	2.458

**Table 13:** Qualitative analysis for 20th order low pass FIR filter.

Algorithm	Minimum stopband attenuation (db)	Minimum stopband attenuation	Execution time (sec)
GWO	− 32.28	0.04	0.4714
PSO	− 14.09	0.61	2.9874
GAO	− 16.37	0.38	3.457
CSO	− 10.47	0.70	0.5985
MFO	− 14.24	0.77	1.214
WOA	− 28.69	0.47	2.24
BBA	− 15.42	0.98	2.101

**Figure 8:**
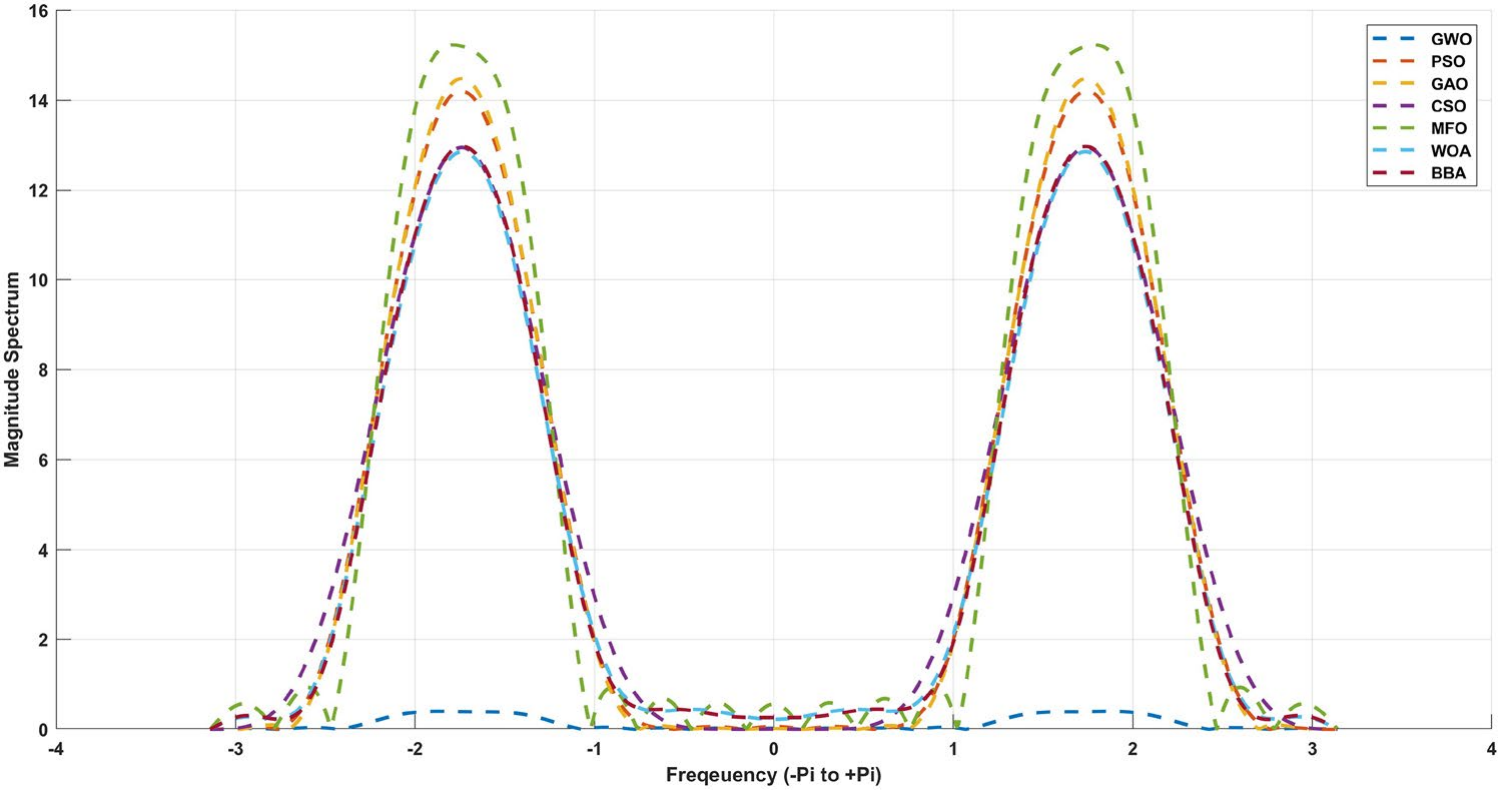
Magnitude response for the 21st order FIR Band-stop filter.

**Figure 9:**
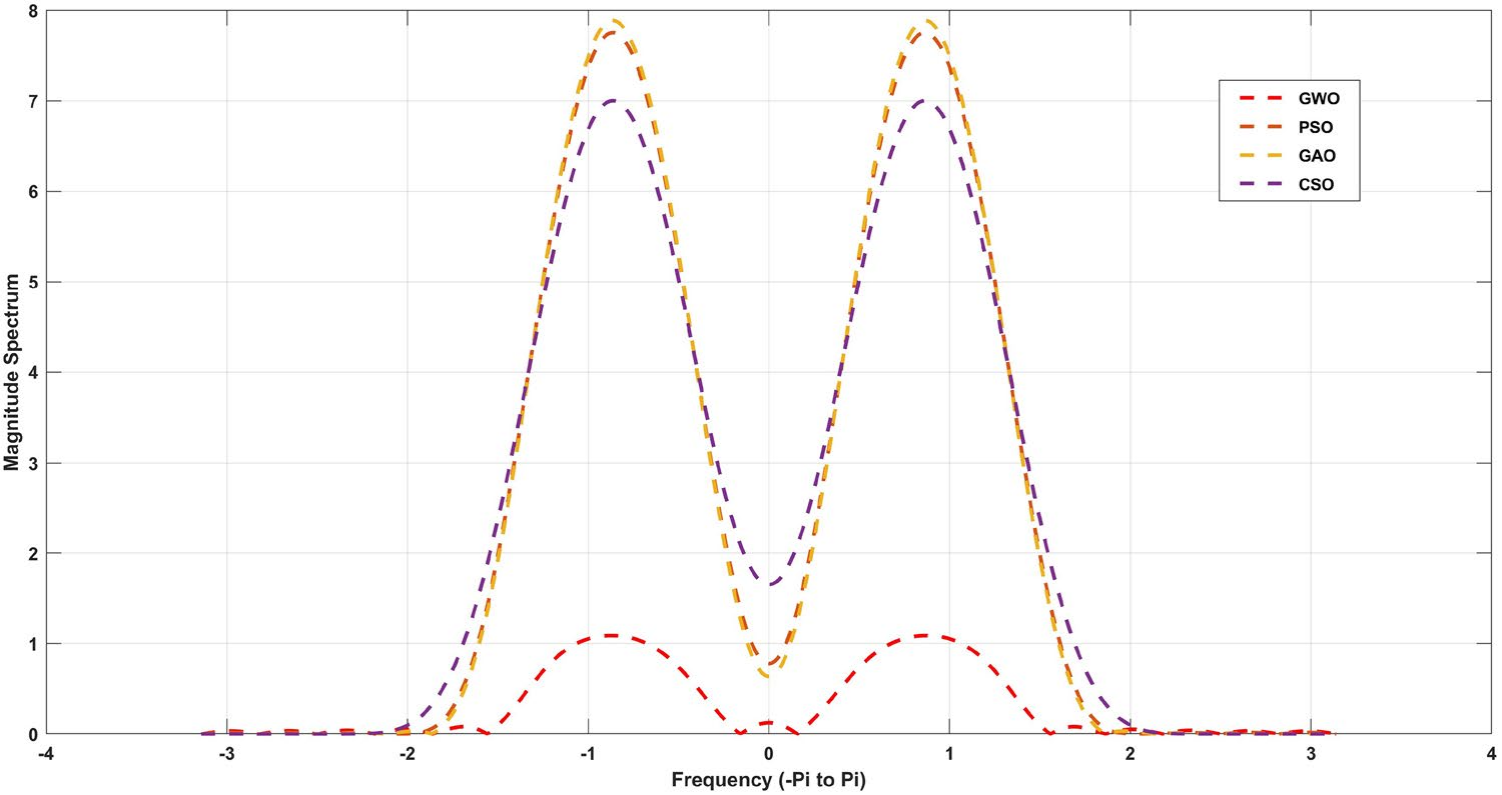
Magnitude response for the 20th order FIR Band-stop filter.

The Fig. 8 for 20th order and 9 for 21st order for 21st order shows a graphical comparison of the magnitude response of the designed FIR low pass filter. These measures are given in Table 12 for 20th order and 13 for 21st order FIR band-stop pass filters. From this Table 13, its observed that a GWO produces the minimum stopband attenuation of − 28.65dB in comparison to PSO (− 20.37dB), GAO (− 15.05dB) and CSO (− 17.52dB). However, the maximum passband attenuation of GWO is 0.05dB, which is minimum from all the other method and same for Table 12 also. In Tables 14 and 15 shows stop-band attenuation coefficients of the designed FIR band-stop pass filter. As seen in 14, The fact that the mean value in the GWO-based FIR band-stop pass filter is very low indicates that the performance of the stopband region is consistent, and the same for the Table 15. In Figs. 8 and 9, the magnitude response of the designed filter is shown. From these figures, GWO shows better performance with very less discontinuity of the FIR filter whereas PSO, GAO and CSO has the moderate discontinuity and almost all the optimization has same magnitude of overshoot. The maximum normalized passband ripple of FIR band-stop pass filter of GWO is 1.357 for 21st order filter and 1.496 for 20th order filter, which makes an overshoot of 0.0046% above the ideal response.

**Table 14:** Quantitative analysis for 21st order band-stop FIR filter.

Algorithm	Maximum	Mean	Variance	Standard deviation
GWO	1.3570	1.1398	0.0077	0.0300
PSO	1.2021	1.0097	0.0068	0.0265
GAO	1.5673	1.3165	0.0089	0.0346
CSO	0.7751	0.6511	0.0044	0.0171
MFO	1.8903	1.5878	0.0107	0.0417
WOA	1.7496	1.4697	0.0099	0.0386
BBA	2.5673	2.1565	0.0146	0.0567

**Table 15:** Quantitative analysis for 20th order band-stop FIR filter.

Algorithm	Maximum	Mean	Variance	Standard deviation
GWO	1.4960	1.2566	0.0085	0.0330
PSO	0.9838	0.8264	0.0056	0.0217
GAO	2.2922	1.9254	0.0130	0.0506
CSO	0.6387	0.5365	0.0036	0.0141
MFO	1.7938	1.5068	0.0102	0.0396
WOA	1.4721	1.2365	0.0084	0.0325
BBA	3.2922	2.7654	0.0187	0.0727

**Figure 10:**
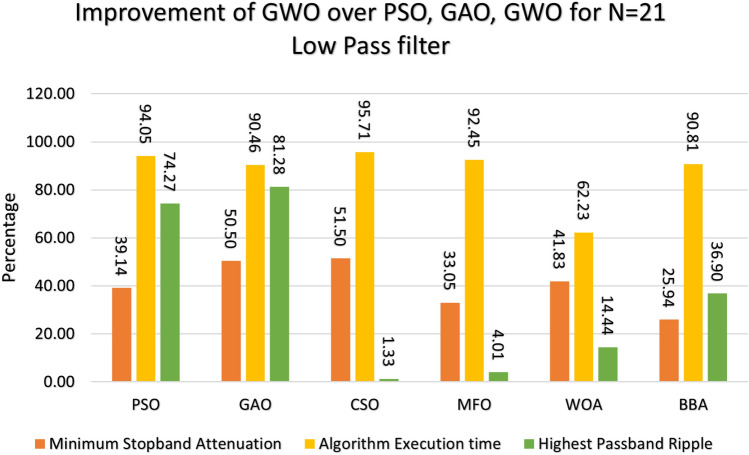
Percentage improvement in GWO compared with other optimization for 20th order low pass filter.

**Figure 11:**
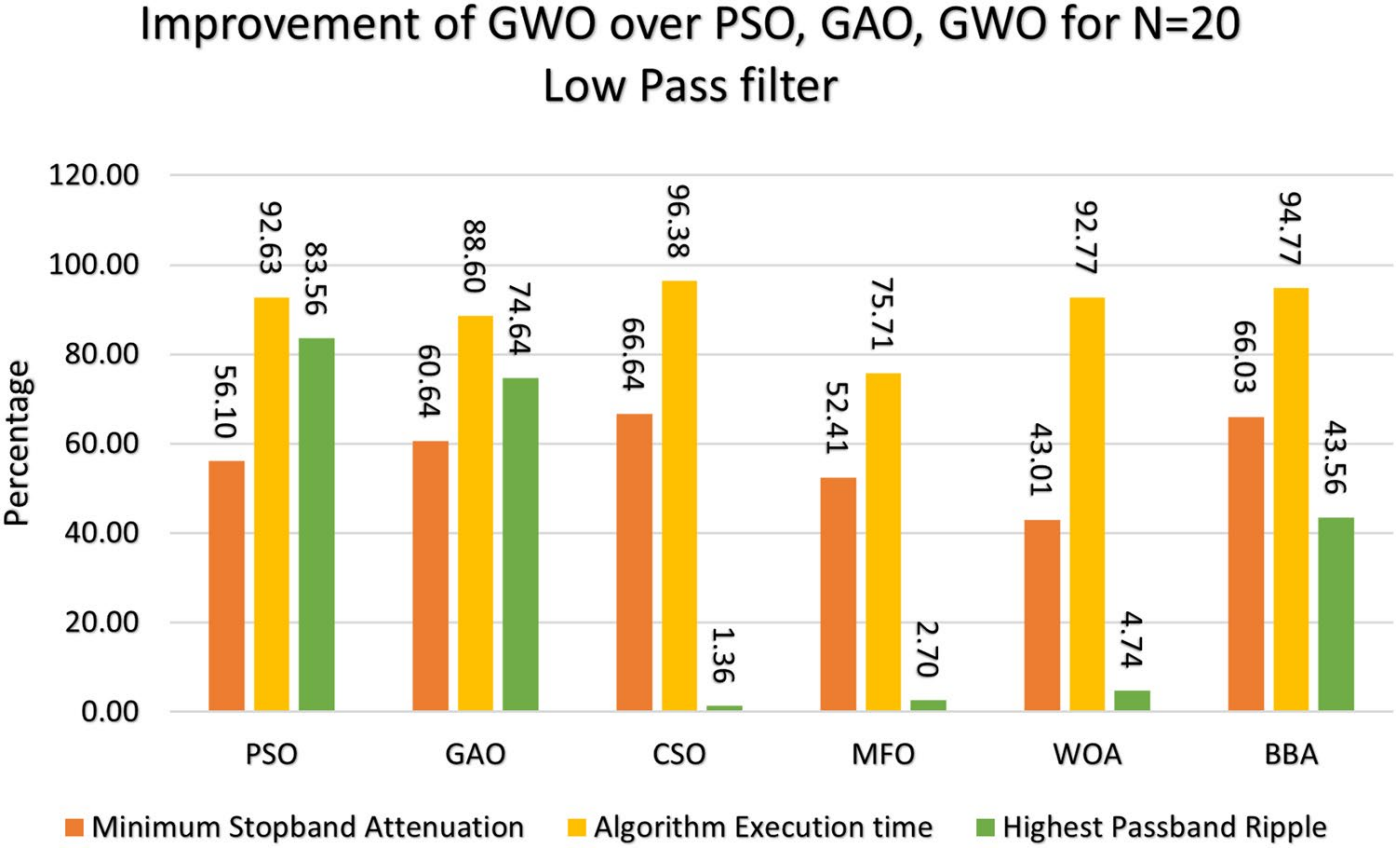
Percentage improvement in GWO compared with other optimization for 21st order low pass filter.

**Figure 12:**
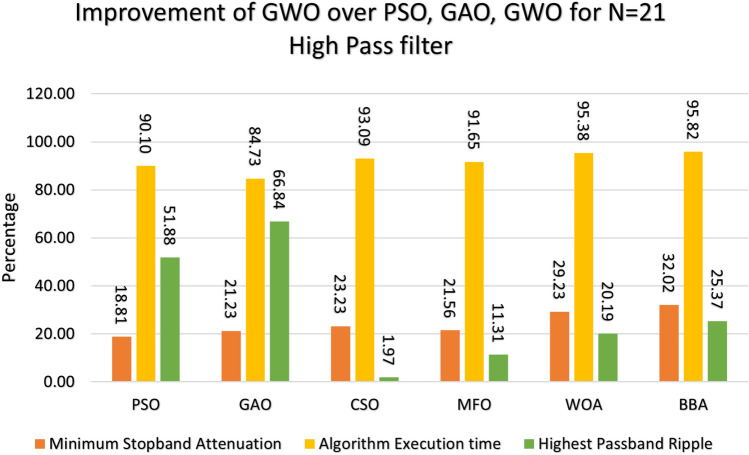
Percentage improvement in GWO compared with other optimization for 2oth order high pass filter.

**Figure 13:**
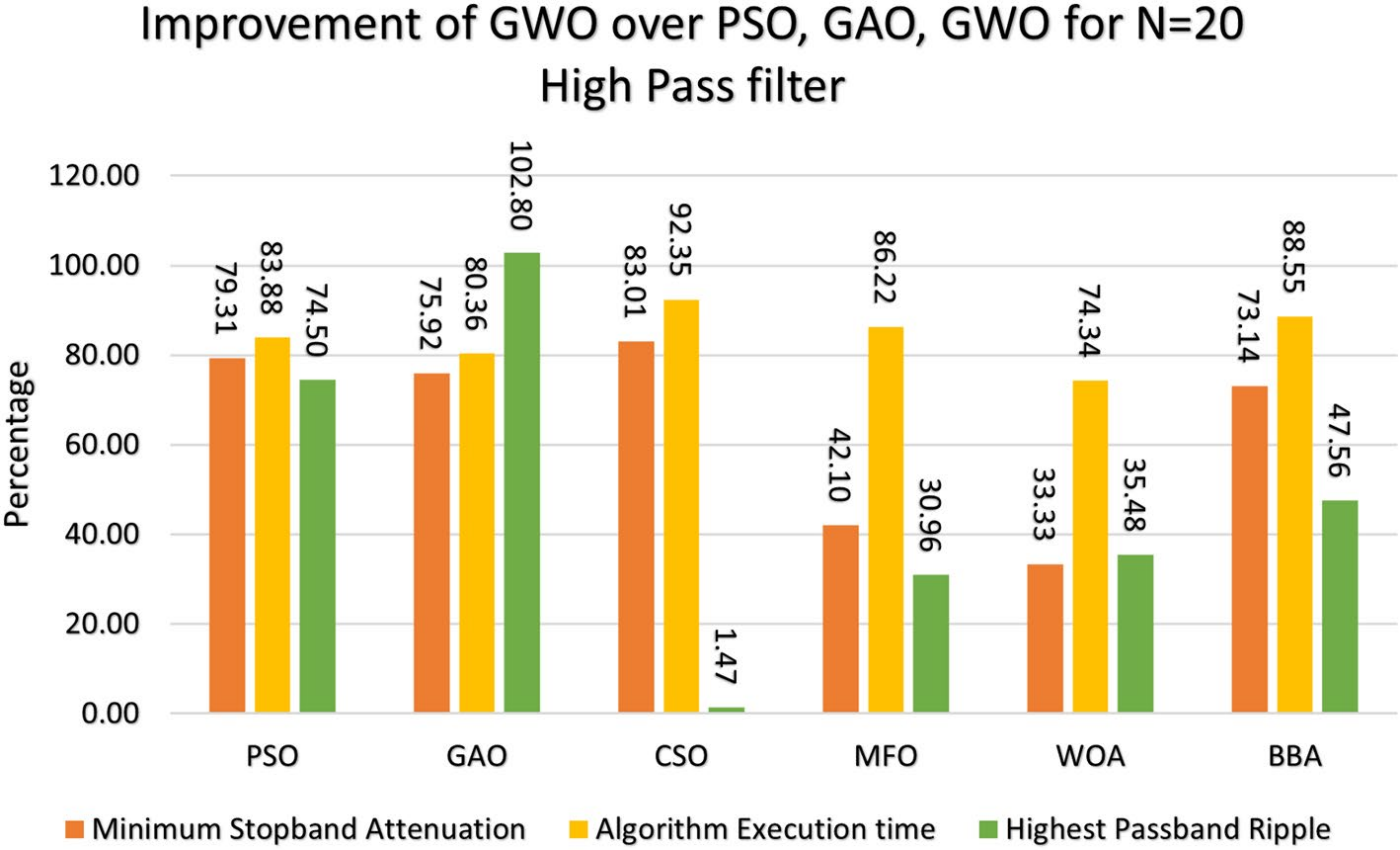
Percentage improvement in GWO compared with other optimization for 21st order high pass filter.

**Figure 14:**
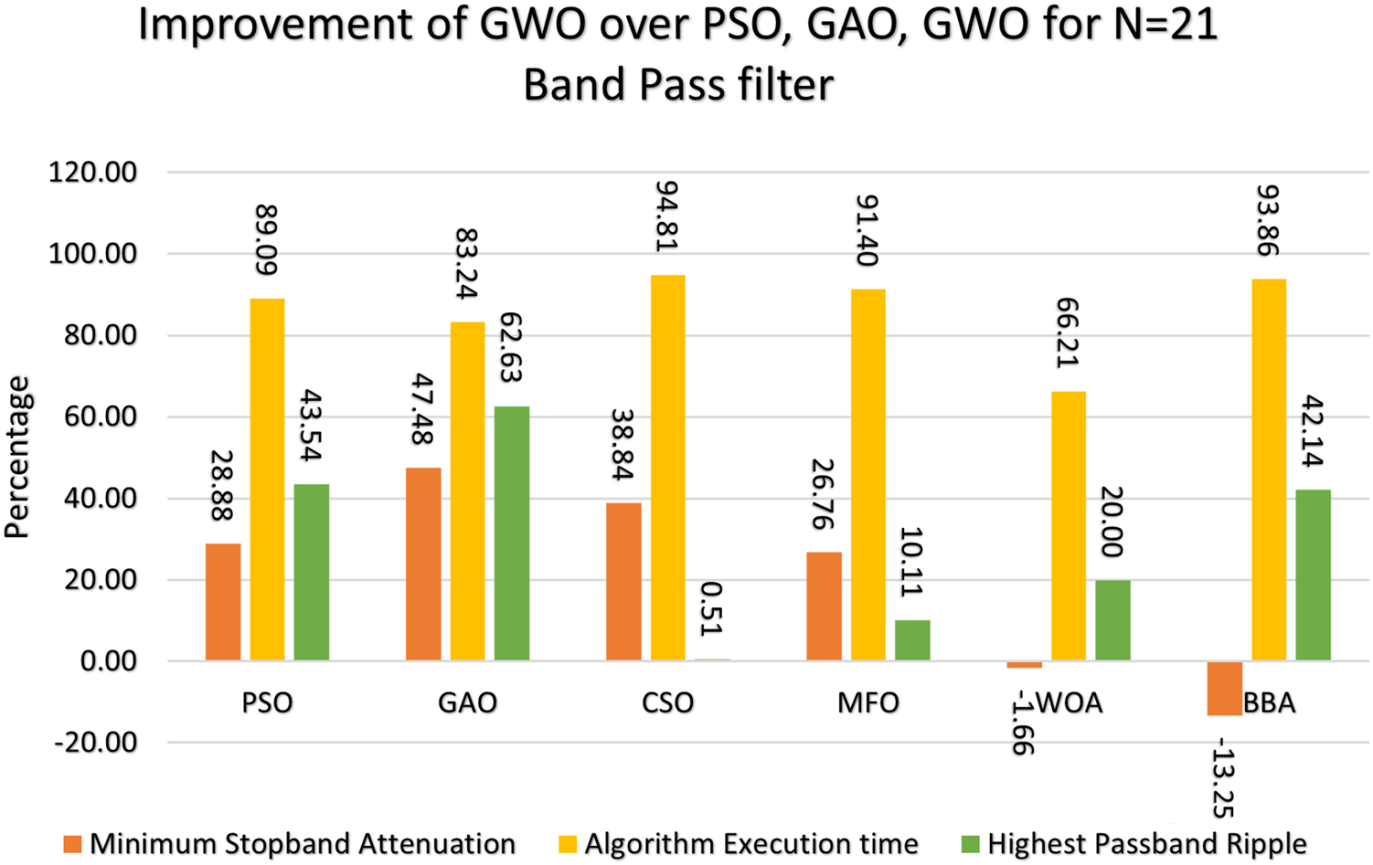
Percentage improvement in GWO compared with other optimization for 20th order Bandpass pass filter.

**Figure 15:**
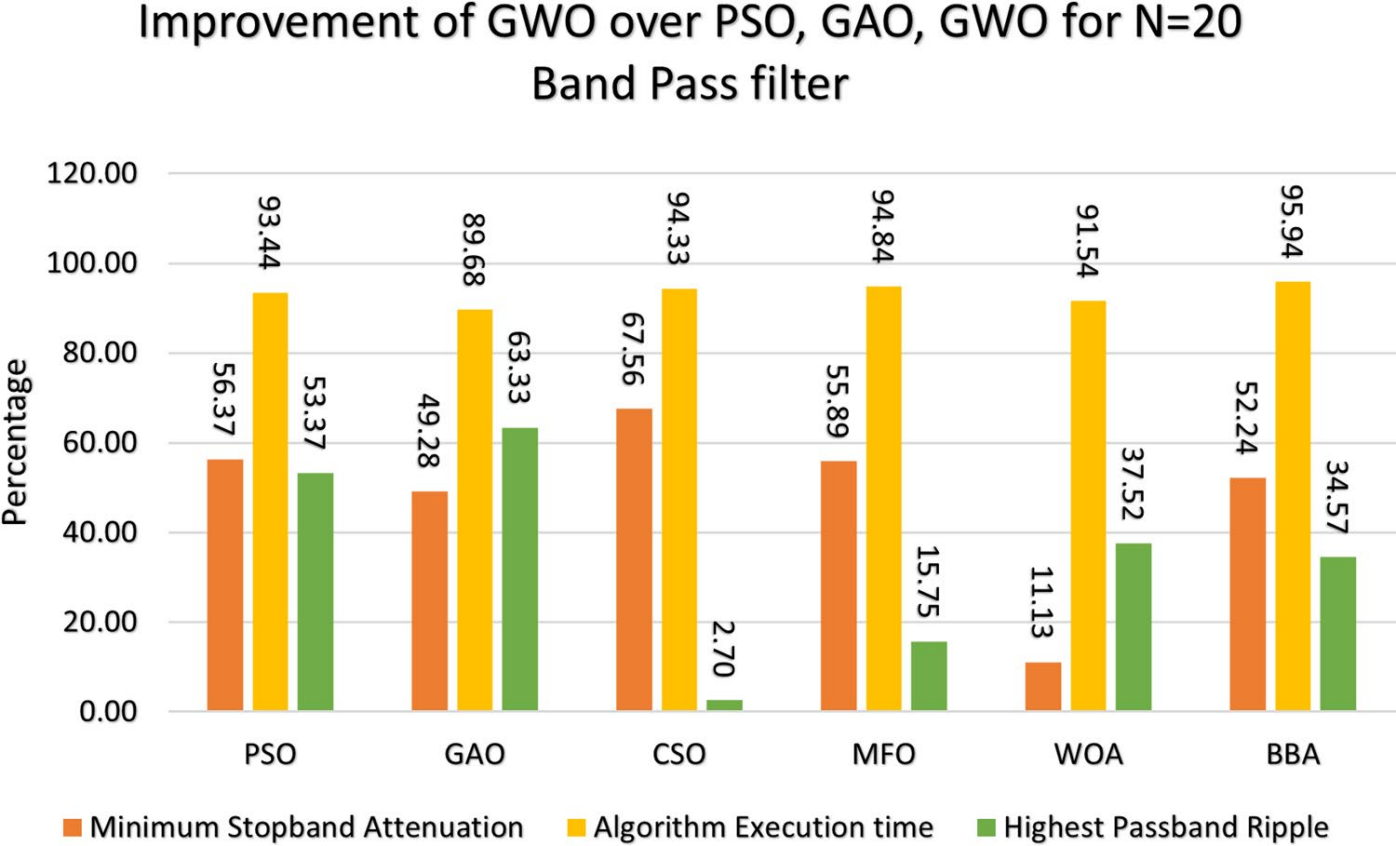
Percentage improvement in GWO compared with other optimization for 21st order Bandpass pass filter.

This section provides a comprehensive analysis of the developed Finite Impulse Response (FIR) filter, highlighting the improvements achieved in the filter design (Low Pass Filter, High Pass Filter, Band Stop Filter) utilizing GWO compared to PSO, GAO, and CSO. The GWO design exhibits a higher percentage improvement in low pass filter characteristics compared to the other designs illustrated in Figs. 10 and 11. The GWO-based lowpass filter coefficients demonstrate a significant enhancement of 39.18%, 88.13%, and 5.93% compared to filters based on PSO, GAO, and CSO, respectively, in terms of achieving the least stopband attenuation. The greatest ripples created demonstrate an improvement of 51.49%, 11.07%, and 5.09% compared to the PSO, GAO, and CSO designs, respectively. Significant progress has been observed in the execution time of the low pass filter design process, as depicted in Figs. 10 and 11. The GWO algorithm converges rapidly and terminates to identify an optimal coefficient. The GWO design exhibits a higher percentage improvement in high pass filter characteristics compared to the other designs illustrated in Figs. 12 and 13. The study demonstrates that the GWO-based lowpass filter coefficients outperform those based on PSO, GAO, and CSO by 18.8%, 83.83%, and 11.80%, respectively, in terms of achieving the smallest stopband attenuation. The greatest ripples created demonstrate an improvement of 23.23%, 16.45%, and 9.7% compared to the PSO, GAO, and CSO designs, respectively. Significant progress has been made in the execution time of the low pass filter design process, as depicted in Fig. 12. The GWO algorithm converges rapidly and terminates when it finds the optimal coefficient. The GWO design exhibits a higher percentage improvement in band pass filter characteristics compared to the other designs illustrated in Fig. 14. The GWO-based lowpass filter coefficients show a significant improvement of 28.88%, 81.32%, and 11.41% compared to filters based on PSO, GAO, and CSO, respectively, in terms of minimal stopband attenuation. The highest ripples achieved demonstrate an improvement of 38.83%, 4.88%, and 42.88% compared to the PSO, GAO, and CSO designs, respectively. Significant progress has been observed in the execution time of the low pass filter design process, as depicted in Fig. 15. The GWO algorithm converges more rapidly and terminates to discover an optimal coefficient.

The results of the study demonstrate a substantial enhancement in the design of HP and BS filters through the utilization of gray wolf optimization techniques. These filters exhibit a high level of accuracy and may be effectively implemented in many applications. The comparative analysis reveals that the proposed filter, developed utilizing the grey wolf optimizer (GWO), exhibits superior performance in comparison to the genetic algorithm optimizer (GAO)^7^, Particle Swarm Optimizer (PSO)^11,23,26^, and Cuckoo search optimizer (CSO), Moth flame (MFO), Whale optimization (WAO) and binary Bat optimization (BBA)^13,24^.

## Conclusion

This study addresses the performance evaluation of constructed finite impulse response (FIR) filters, namely Lowpass, Highpass, and Bandstop filters, utilizing several metaheuristic algorithms, particularly PSO, GAO, CSO, and GWO. The goal of the design is to determine the filter coefficients that minimize the absolute relative error between the filter’s response and the desired output. The solution that was observed demonstrates an effective design that meets the criteria of achieving a high level of attenuation in the stopband and maintaining a uniform response in the passband of a digital filter. To facilitate the design of Lowpass, Highpass, and Bandstop filters, it is possible to employ transformations on the provided design methodology. After conducting a comparative analysis of three optimization algorithms on a standardized platform and with comparable specifications, it was found that the grey wolf optimization (GWO) yielded the most optimal solution. The filter based on the GWO algorithm exhibited the lowest design error and exhibited superior performance in terms of magnitude response, with strong attenuation in the stopband, minimal ripples in the passband and stopband, and a nearly identical transition width. Additionally, it demonstrated the shortest execution time. Moreover, this results in increased flexibility when building the finite impulse response (FIR) filter, since there is no need for meticulous adjustment of parameters. Therefore, it can be inferred that the grey wolf optimization (GWO) is the most optimal heuristic algorithm within the scope of this particular research domain. Presently, our focus lies on the issue of system identification utilizing the grey wolf optimization (GWO) algorithm to maximize outcomes with the objective of achieving a reduction in filter length.

## Data Availability

The datasets used and analysed during the current study available from the corresponding author on reasonable request.
